# Supplementary material for: Discovery and application of insertion-deletion (INDEL) polymorphisms for QTL mapping of early life-history traits in Atlantic salmon
Source: BMC Genomics. 2010 Mar 8;11:156. doi: 10.1186/1471-2164-11-156 (PMC2838853; doi:10.1186/1471-2164-11-156)
Supplement: Additional file 2 — Information on developed 76 locus single-run INDEL panel in Atlantic salmon. Information on fluorescence labeling, primer concentrations, PCR pooling and links to alignments, INDEL motifs and GENESCAN (Burge and Karlin 1997) predictions of genes/exons are available in html format. [file 1471-2164-11-156-S2.ZIP › Additionalfile2/table7444.html]

Contig Alignment

```
Base	A B C D E F G H I J K L M N O P Q R S T U V   
1	A A A . A A . A A A . . . . . . . . . . . .    
2	A A A A A A . A A A . . . . . . . . . . . .    
3	A A A A A A . A A A . . . . . . . . . . . .    
4	C C C C C C . C C C . . . . . . . . . . . .    
5	A A A A A A . A A A . . . . . . . . . . . .    
6	T T T T T T . T T T . . . . . . . . . . . .    
7	A A A A A A . A A A . . . . . . . . . . . .    
8	T T T T T T . T T T . . . . . . . . . . . .    
9	A A A A A A . A A A . . . . . . . . . . . .    
10	T T T T T T . T T T . . . . . . . . . . . .    
11	C C C C C C . C C C . . . . . . . . . . . .    
12	C C C C C C . C C C . . . . . . . . . . . .    
13	G G G G G G . G G G . . . . . . . . . . . .    
14	T T T T T T . T T T . . . . . . . . . . . .    
15	C C C C C C . C C C . . . . . . . . . . . .    
16	T T T T T T . T T T . . . . . . . . . . . .    
17	C C C C C C . C C C . . . . . . . . . . . .    
18	T T T T T T . T T T . . . . . . . . . . . .    
19	A A A A A A . A A A . . . . . . . . . . . .    
20	C C C C C C . C C C . . . . . . . . . . . .    
21	A A A A A A A A A A . . . . . . . . . . . .    
22	C C C C C C C C C C . . . . . . . . . . . .    
23	A A A A A A A A A A . . . . . . . . . . . .    
24	T T T T T T T T T T . . . . . . . . . . . .    
25	G G G G G G G G G G . . . . . . . . . . . .    
26	T T T T T T T T T T . . . . . . . . . . . .    
27	T T T T T T T T T T . . . . . . . . . . . .    
28	G G G G G G G G G G . . . . . . . . . . . .    
29	A A A A A A A A A A . . . . . . . . . . . .    
30	A A A A A A A A A A . . . . . . . . . . . .    
31	A A A A A A A A A A . . . . . . . . . . . .    
32	T T T T T T T T T T . . . . . . . . . . . .    
33	G G G G G G G G G G . . . . . . . . . . . .    
34	T T T T T T T T T T . . . . . . . . . . . .    
35	T T T T T T T T T T . . . . . . . . . . . .    
36	G G G G G G G G G G . . . . . . . . . . . .    
37	G G G G G G G G G G . . . . . . . . . . . .    
38	C C C C C C C C C C . . . . . . . . . . . .    
39	G G G G G G G G G G . . . . . . . . . . . .    
40	T T T T T T T T T T . . . . . . . . . . . .    
41	T T T T T T T T T T . . . . . . . . . . . .    
42	T T T T T T T T T T . . . . . . . . . . . .    
43	C C C C C C C C C C . . . . . . . . . . . .    
44	G G G G G G G G G G . . . . . . . . . . . .    
45	C C C C C C C C C C . . . . . . . . . . . .    
46	T T T T T T T T T T . . . . . . . . . . . .    
47	G G G G G G G G G G . . . . . . . . . . . .    
48	C C C C C C C C C C . . . . . . . . . . . .    
49	T T T T T T T T T T . . . . . . . . . . . .    
50	G G G G G G G G G G . . . . . . . . . . . .    
51	G G G G G G G G G G . . . . . . . . . . . .    
52	T T T T T T T T T T . . . . . . . . . . . .    
53	G G G G G G G G G G . . . . . . . . . . . .    
54	A A A A A A A A A A . . . . . . . . . . . .    
55	G G G G G G G G G G . . . . . . . . . . . .    
56	A A A A A A A A A A . . . . . . . . . . . .    
57	G G G G G G G G G G . . . . . . . . . . . .    
58	G G G G G G G G G G . . . . . . . . . . . .    
59	G G G G G G G G G G . . . . . . . . . . . .    
60	A A A A A A A A A A . . . . . . . . . . . .    
61	C C C C C C C C C C . . . . . . . . . . . .    
62	A A A A A A A A A A . . . . . . . . . . . .    
63	C C C C C C C C C C . . . . . . . . . . . .    
64	T T T T T T T T T T . . . . . . . . . . . .    
65	T T T T T T T T T T . . . . . . . . . . . .    
66	C C C C C C C C C C . . . . . . . . . . . .    
67	G G G G G G G G G G . . . . . . . . . . . .    
68	A A A A A A A A A A . . . . . . . . . . . .    
69	T T T T T T T T T T . . . . . . . . . . . .    
70	A A A A A A A A A A . . . . . . . . . . . .    
71	G G G G G G G G G G . . . . . . . . . . . .    
72	T T T T T T T T T T . . . . . . . . . . . .    
73	C C C C C C C C C T . . . . . . . . . . . .    
74	T T T T T T T T T T . . . . . . . . . . . .    
75	T T T T T T T T T T . . . . . . . . . . . .    
76	T T T T T T T T T T . . . . . . . . . . . .    
77	G G G G G G G G G G . . . . . . . . . . . .    
78	A A A A A A A A A A . . . . . . . . . . . .    
79	A A A A A A A A A A . . . . . . . . . . . .    
80	A A A A A A A A A A . . . . . . . . . . . .    
81	A A A A A A A A A A . . . . . . . . . . . .    
82	T T T T T T T T T T . . . . . . . . . . . .    
83	A A A A A A A A A A . . . . . . . . . . . .    
84	A A A A A A A A A A . . . . . . . . . . . .    
85	T T T T T T T T T T . . . . . . . . . . . .    
86	A A A A A A A A A A . . . . . . . . . . . .    
87	T T T T T T T T T T . . . . . . . . . . . .    
88	T T T T T T T T T T . . . . . . . . . . . .    
89	T T T T T T T T T T . . . . . . . . . . . .    
90	G G G G G G G G G G . . . . . . . . . . . .    
91	A A A A A A A A A A . . . . . . . . . . . .    
92	G G G G G G G G G G . . . . . . . . . . . .    
93	C C C C C C C C C C . . . . . . . . . . . .    
94	A A A A A A A A A A . . . . . . . . . . . .    
95	C C C C C C C C C C . . . . . . . . . . . .    
96	T T T T T T T T T T . . . . . . . . . . . .    
97	T T T T T T T T T T . . . . . . . . . . . .    
98	C C C C C C C C C C . . . . . . . . . . . .    
99	T T T T T T T T T T . . . . . . . . . . . .    
100	G G G G G G G G G G . . . . . . . . . . . .    
101	G G G G G G G G G G . . . . . . . . . . . .    
102	G G G G G G G G G G . . . . . . . . . . . .    
103	G G G G G G G G G G . . . . . . . . . . . .    
104	T G G T G G G G T G . . . . . . . . . . . .    
105	T T T T T T T T T T . . . . . . . . . . . .    
106	T T T T T T T T T T . . . . . . . . . . . .    
107	T T T T T T T T T T . . . . . . . . . . . .    
108	T T T T T T T T T T . . . . . . . . . . . .    
109	T T T T T T T T T T . . . . . . . . . . . .    
110	T T T T T T T T T T . . . . . . . . . . . .    
111	C C C C C C C C C C . . . . . . . . . . . .    
112	T T T T T T T T T T . . . . . . . . . . . .    
113	C C C C C C C C C C . . . . . . . . . . . .    
114	C C C C C C C C C C . . . . . . . . . . . .    
115	A A A A A A A A A A . . . . . . . . . . . .    
116	A A A A A A A A A A . . . . . . . . . . . .    
117	A A A A A A A A A A . . . . . . . . . . . .    
118	A A A A A A A A A A . . . . . . . . . . . .    
119	G G G C G G G G G G . . . . . . . . . . . .    
120	C C C C C C C C C C . . . . . . . . . . . .    
121	A A A G A A A A A A . . . . . . . . . . . .    
122	A A A A A A A A A A . . . . . . . . . . . .    
123	C C C C C C C C C C . . . . . . . . . . . .    
124	A A A A A A A A A A . . . . . . . . . . . .    
125	A A A A A A A A A A . . . . . . . . . . . .    
126	C C C C C C C C C C . . . . . . . . . . . .    
127	A A A A A A A A A A . . . . . . . . . . . .    
128	A A A A A A A A A A . . . . . . . . . . . .    
129	T T T T T T T T T T . . . . . . . . . . . .    
130	G G G G G G G G G G . . . . . . . . . . . .    
131	A A A A A A A A A A . . . . . . . . . . . .    
132	T T T T T T T T T T . . . . . . . . . . . .    
133	A A A A A A A A A A . . . . . . . . . . . .    
134	A A A A A A A A A A . . . . . . . . . . . .    
135	A A A A A A A A A A . . . . . . . . . . . .    
136	A A A A A A A A A A . . . . . . . . . . . .    
137	C C C C C C C C C C . . . . . . . . . . . .    
138	A A A A A A A A A A . . . . . . . . . . . .    
139	T T T T T T T T T T . . . . . . . . . . . .    
140	A A A T A A A A A A . . . . . . . . . . . .    
141	T T T T T T T T T T . . . . . . . . . . . .    
142	G G G G G G G G G G . . . . . . . . . . . .    
143	T T T T T T T T T T . . . . . . . . . . . .    
144	C C C C C C C C C C . . . . . . . . . . . .    
145	A A A A A A A A A A . . . . . . . . . . . .    
146	A A A A A A A A A A . . . . . . . . . . . .    
147	C C C C C C C C C C . . . . . . . . . . . .    
148	A A A A A A A A A A . . . . . . . . . . . .    
149	A A A A A A A A A A . . . . . . . . . . . .    
150	A A A A A A A A A A . . . . . . . . . . . .    
151	C C C C C C C C C C . . . . . . . . . . . .    
152	A A A A A A A A A A . . . . . . . . . . . .    
153	C C C C C C C C C C . . . . . . . . . . . .    
154	A A A A A A A A A A . . . . . . . . . . . .    
155	A A A A A A A A A A . . . . . . . . . . . .    
156	A A A A A A A A A A . . . . . . . . . . . .    
157	A A A A A A A A A A . . . . . . . . . . . .    
158	T T T T T T T T T T . . . . . . . . . . . .    
159	G G G G G G G G G G . . . . . . . . . . . .    
160	A A A A A A A A A A . . . . . . . . . . . .    
161	A A A A A A A A A A . . . . . . . . . . . .    
162	A A A A A A A A A A . . . . . . . . . . . .    
163	T T T T T T T T T T . . . . . . . . . . . .    
164	G G G G G G G G G G . . . . . . . . . . . .    
165	T T T T T T T T T T . . . . . . . . . . . .    
166	C C C C C C C C C C . . . . . . . . . . . .    
167	A A A G A A A A A A . . . . . . . . . . . .    
168	A A A A A A A A A A . . . . . . . . . . . .    
169	G G G G G G G G G G . . . . . . . . . . . .    
170	C C C C C C C C C C . . . . . . . . . . . .    
171	T T T T T T T T T T . . . . . . . . . . . .    
172	A A A A A A A A A A . . . . . . . . . . . .    
173	A A A A A A A A A A . . . . . . . . . . . .    
174	A A A A A A A A A A . . . . . . . . . . . .    
175	A A A A A A A A A A . . . . . . . . . . . .    
176	A A A A A A A A A A . . . . . . . . . . . .    
177	A A A A A A A A A A . . . . . . . . . . . .    
178	G G G G A G G G G G . . . . . . . . . . . .    
179	T T T T T T T T T T . . . . . . . . . . . .    
180	A A A A A A A A A A . . . . . . . . . . . .    
181	C C C C C C C C C C . . . . . . . . . . . .    
182	A A A A A A A A A A . . . . . . . . . . . .    
183	A A A A A A A A A A . . . . . . . . . . . .    
184	T T T T T T T T T T . . . . . . . . . . . .    
185	T T T T T T T T T T . . . . . . . . . . . .    
186	C C C C C C C C C C . . . . . . . . . . . .    
187	T T T T T T T T T T . . . . . . . . . . . .    
188	C C C C C C C C C C . . . . . . . . . . . .    
189	T T T T T T T T T T . . . . . . . . . . . .    
190	C C C C C C C C C C . . . . . . . . . . . .    
191	T T T T T T T T T T . . . . . . . . . . . .    
192	A A A A A A A A A A . . . . . . . . . . . .    
193	G G G G G G G G G G . . . . . . . . . . . .    
194	T T T T T T T T T T . . . . . . . . . . . .    
195	A A A A A A A A A A . . . . . . . . . . . .    
196	C C C C C C C C C C . . . . . . . . . . . .    
197	A A A A A A A A A A . . . . . . . . . . . .    
198	T T T T T T T T T T . . . . . . . . . . . .    
199	C C C C C C C C C C . . . . . . . . . . . .    
200	C C C C C C C C C C . . . . . . . . . . . .    
201	G G G G G G G G G G . . . . . . . . . . . .    
202	G G G G G G G G G G . . . . . . . . . . . .    
203	G G G G G G G G G G . . . . . . . . . . . .    
204	A A A A A A A A A A . . . . . . . . . . . .    
205	T T T T T T T T T T . . . . . . . . . . . .    
206	C C C C C C C C C C . . . . . . . . . . . .    
207	C C C C C C C C C C . . . . . . . . . . . .    
208	A A A A A A A A A A . . . . . . . . . . . .    
209	T T T T T T T T T T . . . . . . . . . . . .    
210	G G G G G G G G G G . . . . . . . . . . . .    
211	G G G G G G G G G G . . . . . . . . . . . .    
212	A A A A A A A A A A . . . . . . . . . . . .    
213	T T T T T T T T T T . . . . . . . . . . . .    
214	C C C C C C C C C C . . . . . . . . . . . .    
215	T T T T T T T T T T . . . . . . . . . . . .    
216	G G G G G G G G G G . . . . . . . . . . . .    
217	A A A A A A A A A A . . . . . . . . . . . .    
218	A A A A A A A A A A . . . . . . . . . . . .    
219	A A A A A A A A A A . . . . . . . . . . . .    
220	A A A A A A A A A A . . . . . . . . . . . .    
221	A A A A A A A A A A . . . . . . . . . . . .    
222	G G G G G G G G G G . . . . . . . . . . . .    
223	C C C C C C C C C C . . . . . . . . . . . .    
224	C C C C C C C C C C . . . . . . . . . . . .    
225	A A A A A A A A A A . . . . . . . . . . . .    
226	T T T T T T T T T T . . . . . . . . . . . .    
227	T T T T T T T T T T . . . . . . . . . . . .    
228	C C C C C C C C C C . . . . . . . . . . . .    
229	A A A A A A A A A A . . . . . . . . . . . .    
230	C C C C C C C C C C . . . . . . . . . . . .    
231	G G G G G G G G G G . . . . . . . . . . . .    
232	T T T T T T T T T T . . . . . . . . . . . .    
233	C C C C C C C C C C . . . . . . . . . . . .    
234	T T T T T T T T T T . . . . . . . . . . . .    
235	T T T T T T T T T T . . . . . . . . . . . .    
236	T T T T T T T T T T . . . . . . . . . . . .    
237	G G G G G G G G G G . . . . . . . . . . . .    
238	G G G G G G G G G G . . . . . . . . . . . .    
239	A A A A A A A A A A . . . . . . . . . . . .    
240	A A A A A A A A A C . . . . . . . . . . . .    
241	C C C C C C C C C C . . . . . . . . . . . .    
242	A A A A A A A A A A . . . . . . . . . . . .    
243	C C C C C C C C C C . . . . . . . . . . . .    
244	T T T T T T T T T T . . . . . . . . . . . .    
245	G G G G G G G G G G . . . . . . . . . . . .    
246	A A A A A A A A A A . . . . . . . . . . . .    
247	C C C C C C C C C C . . . . . . . . . . . .    
248	A A A G A A A A A A . . . . . . . . . . . .    
249	C C C C C C C C C C . . . . . . . . . . . .    
250	A A A A A A A A A A . . . . . . . . . . . .    
251	A A A A A A A A A A . . . . . . . . . . . .    
252	G G G G G G G G G G . . . . . . . . . . . .    
253	A A A A A A A A A A . . . . . . . . . . . .    
254	C C C C C C C C C C . . . . . . . . . . . .    
255	T T T T T T T T T T . . . . . . . . . . . .    
256	G G G G G G G G G G . . . . . . . . . . . .    
257	T T T C T T T T T T . . . . . . . . . . . .    
258	C C C C C C C C C C . . . . . . . . . . . .    
259	A A A A A A A A A A . . . . . . . . . . . .    
260	T T T T T T T T T T . . . . . . . . . . . .    
261	T T T T T T T T T T . . . . . . . . . . . .    
262	C C C C C C C C C C . . . . . . . . . . . .    
263	C C C C C C C C C C . . . . . . . . . . . .    
264	C C C C C C C C C C . . . . . . . . . . . .    
265	T T T T T T T T T T . . . . . . . . . . . .    
266	T T T T T T T T T T . . . . . . . . . . . .    
267	A A A A A A A A A A . . . . . . . . . . . .    
268	T T T T T T T T T T . . . . . . . . . . . .    
269	C C C C C C C C C C . . . . . . . . . . . .    
270	C C C C C C C C C C . . . . . . . . . . . .    
271	A A A A A A A A A A . . . . . . . . . . . .    
272	C C C C C C C C C C . . . . . . . . . . . .    
273	T T T T T T T T T T . . . . . . . . . . . .    
274	C C C C C C C C C C . . . . . . . . . . . .    
275	A A A A A A A A A A . . . . . . . . . . . .    
276	T T T T T T T T T T . . . . . . . . . . . .    
277	T T T T T T T T T T . . . . . . . . . . . .    
278	C C C C C C C C C C . . . . . . . . . . . .    
279	G G G G G G G G G G . . . . . . . . . . . .    
280	G G G G G G G G G G . . . . . . . . . . . .    
281	G G G G G G G G G G . . . . . . . . . . . .    
282	G G G G G G G G G G . . . . . . . . . . . .    
283	T T T T T T T T T T . . . . . . . . . . . .    
284	G G G G G G G G G G . . . . . . . . . . . .    
285	G G G G G G G G G G . . . . . . . . . . . .    
286	T T T T T T T T T T . . . . . . . . . . . .    
287	A A A A A A A A A A . . . . . . . . . . . .    
288	T T T T T T T T T T . . . . . . . . . . . .    
289	T T T T T T T T T T . . . . . . . . . . . .    
290	T T T T T T T T T T . . . . . . . . . . . .    
291	C C C C C C C C C C . . . . . . . . . . . .    
292	T T T T T T T T T T . . . . . . . . . . . .    
293	C C C C C C C C C C . . . . . . . . . . . .    
294	T T T T T T T T T T . . . . . . . . . . . .    
295	C C C C C C C C C C . . . . . . . . . . . .    
296	T T T T T T T T T T . . . . . . . . . . . .    
297	C C C C C C C C C C . . . . . . . . . . . .    
298	C C C C C C C C C C . . . . . . . . . . . .    
299	T T T T T T T T T T . . . . . . . . . . . .    
300	C C C C C C C C C C . . . . . . . . . . . .    
301	C C C C C C C C C C . . . . . . . . . . . .    
302	C C C C C C C C C C . . . . . . . . . . . .    
303	T T T T T T T T T T . . . . . . . . . . . .    
304	C C C C C C C C C C . . . . . . . . . . . .    
305	C C C C C C C C C C . . . . . . . . . . . .    
306	A A A G A A A A A A . . . . . . . . . . . .    
307	C C C C C C C C C C . . . . . . . . . . . .    
308	C C C C C C C C C C . . . . . . . . . . . .    
309	T T T T T T T T T T . . . . . . . . . . . .    
310	C C C C C C C C C C . . . . . . . . . . . .    
311	A A A A A A A A A A . . . . . . . . . . . .    
312	T T T T T T T T T T . . . . . . . . . . . .    
313	C C C C C C C C C C . . . . . . . . . . . .    
314	C C C C C C C C C C . . . C . . . . . . . .    
315	T T T T T T T T T T . . . T . . . . . . . .    
316	G G G G G G G G G G . . . G . . . . . . . .    
317	C C C C C C C C C C . . . C . . . . . . . .    
318	G G G G G G G G G G . . . G . . . . . . . .    
319	C C C C C C C C C C . . . C . . . . . . . .    
320	T T T T T T T T T T . . . T . . . . . . . .    
321	C C C C C C C C C C . . . C . . . . . . . .    
322	C C C C C C C C C C . . . C . . . . . . . .    
323	T T T T T T T T T T . . . T . . . . . . . .    
324	T T T T T T T T T T . . . T . . . . . . . .    
325	T T T T T T T T T T . . . T . . . . . . . .    
326	C C C C C C C C C C . . . C . . . . . . . .    
327	T T T T T T T T T T . . . T . . . . . . . .    
328	C C C C C C C C C C . . . C . . . . . . . .    
329	C C C C C C C C C C . . . C . . . . . . . .    
330	T T T T T T T T T T . . . T . . . . . . . .    
331	G G G G G G G G G G . . . G . . . . . . . .    
332	C C C C C C C C C C . . . C . . . . . . . .    
333	T T T T T T T T T T . . . T . . . . . . . .    
334	G G G G G G G G G G . . . G . . . . . . . .    
335	C C C C C C C C C C . . . C . . . . . . . .    
336	A A A A A A A A A A . . . A . . . . . . . .    
337	C C C C C C C C C C . . . C . . . . . . . .    
338	C C C C C C C C C C . . . C . . . . . . . .    
339	T T T T T T T T T T . . . T . . . . . . . .    
340	C C C C C C C C C C . . . C . . . . . . . .    
341	T T T T T T T T T T . . . T . . . . . . . .    
342	G G G G G G G G G G . . . G . . . . . . . .    
343	A A A A A A A A A A . . . A . . . . . . . .    
344	A A A A A A A A A A . . . A . . . . . . . .    
345	G G G G G G G G G G . . . G . . . . . . . .    
346	C C C C C C C C C C . . . C . . . . . . . .    
347	C C C C C C C C C C . . C C . . . . . . . .    
348	T T T T T T T T T T . . T T . . . . . . . .    
349	T T T T T T T T T T . . T T . . . . . . . .    
350	G G G G G G G G G G . . G G . . . . . . . .    
351	G G G G G G G G G G . . G G . . . . . . . .    
352	T T T T T T T T T T . . T T . . . . . . . .    
353	C C C C C C C C C C . . C C . . . . . . . .    
354	T T T T T T T T T T . . T T . . . . . . . .    
355	T T T T T T T T T T . . T T . . . . . . . .    
356	G G G G G G G G G G . . G G . . . . . . . .    
357	T T T T T T T T T T . . T T . . . . . . . .    
358	G G G G G G G G G G . . G G . . . . . . . .    
359	G G G G G G G G G G . . G G . . . . . . . .    
360	C C C C C C C C C C . . C C . . . . . . . .    
361	T T T T T T T T T T . . T T . . . . . . . .    
362	C C C C C C C C C C . . C C . . . . . . . .    
363	T T T T T T T T T T . . T T . . . . . . . .    
364	G G G G G G G G G G . . G G . . . . . . . .    
365	T T T T T T T T T T . . T T . . . . . . . .    
366	T T T T T T T T T T . . T T . . . . . . . .    
367	C C C C C C C C C C . . C C C . . . . . . .    
368	G G G G G G G G G G . . G G G . . . . . . .    
369	G G G G G G G G G G . . G G G . . . . . . .    
370	T T T T T T T T T T . . T T T . . . . . . .    
371	G G G G G G G G G G . . G G G . . . . . . .    
372	C C C C C C C C C C . . C C C . . . . . . .    
373	C C C C C C C C C C . . C C C . . . . . . .    
374	C C C C C C C C C C . . C C C . . . . . . .    
375	A A A A A A A A A A . . A A A . . . . . . .    
376	G G G G G G G G G G . . G G G . . . . . . .    
377	G G G G G G G G G G . . G G G . . . . . . .    
378	C C C C C C C C C C . . C C C . . . . . . .    
379	C C C C C C C C C C . . C C C . . . . . . .    
380	T T T T T T T T T T . . T T T . . . . . . .    
381	G G G G G G G G G G . . G G G . . . . . . .    
382	A A A A A A A A A A . . A A A . . . . . . .    
383	C C C C C C C C C C . . C C C . . . . . . .    
384	C C C C C C C C C C . . C C C . . . . . . .    
385	C C C C C C C C C C . . C C C . . . . . . .    
386	A A A A A A A A A A . . A A A . . . . . . .    
387	G G G G G G G G G G . . G G G . . . . . . .    
388	G G G G G G G G G G . . G G G . . . . . . .    
389	T T T T T T T T T T . . T T T . . . . . . .    
390	G G G G G G G G G G . . G G G . . . . . . .    
391	T T T T T T T T T T . . T T T . . . . . . .    
392	G G G G G G G G G G . . G G G . . . . . . .    
393	T T T T T T T T T T . . T T T . . . . . . .    
394	G G G G G G G G G G . . G G G . . . . . . .    
395	T T T T T T T T T T . . T T T . . . . . . .    
396	A A A A A A A A A A . . A A A . . . . . . .    
397	G G G G G G G G G G . . G G G . . . . . . .    
398	C C C C C C C C C C . . C C C . . . . . . .    
399	T T T T T T T T T T . . T T T . . . . . . .    
400	C C C C C C C C C C . . C C C . . . . . . .    
401	C C C C C C C C C C . . C C C . . . . . . .    
402	T T T T T T T T T T . . T T T . . . . . . .    
403	T T T T T T T T T T . . T T T . . . . . . .    
404	G G G G G G G G G G . . G G G . . . . . . .    
405	G G G G G G G G G G . . G G G . . . . . . .    
406	A A A A A A A A A A . . A A A . . . . . . .    
407	C C C C C C C C C C . . C C C . . . . . . .    
408	A A A A A A A A A A . . A A A . . . . . . .    
409	C C C C C C C C C C . . C C C . . . . . . .    
410	C C C C C C C C C C . . C C C . . . . . . .    
411	A A A A A A A A A A . . A A A . . . . . . .    
412	C C C C C C C C C C . . C C C . . . . . . .    
413	A A A A A A A A A A . . A A A . . . . . . .    
414	C C C C C C C C C C . . C C C . . . . . . .    
415	A A A A A A A A A A . . A A A . . . . . . .    
416	C C C C C C C C C C . . C C C . . . . . . .    
417	A A A A A A A A A A . . A A A . . . . . . .    
418	G G G G G G G G G G . . G G G . . . . . . .    
419	A A A A A A A A A A . . A A A . . . . . . .    
420	A A A A A A A A A A . . A A A . . . . . . .    
421	A A A A A A A A A A . . A A A . . . . . . .    
422	C C C C C C C C C C . . C C C . . . . . . .    
423	T T T T T T T T T T . . T T T . . . . . . .    
424	C C C C C C C C C C . . C C C . . . . . . .    
425	T T T T T T T T T T . . T T T . . . . . . .    
426	G G G G G G G G G G . . G G G . . . . . . .    
427	C C C C C C C C C C . . C C C . . . . . . .    
428	C C C C C C C C C C . . C C C . . . . . . .    
429	T T T T T T T T T T . . T T T . . . . . . .    
430	G G G G G G G G G G . . G G G . . . . . . .    
431	G G G G G G G G G G . . G G G . . . . . . .    
432	T T T T T T T T T T . . T T T . . . . . . .    
433	C C C C C C C C C C . . C C C . . . . . . .    
434	C C C C C C C C C C . . C C C . . . . . . .    
435	T T T T T T T T T T . . T T T . . . . . . .    
436	C C C C C C C C C C . . C C C . . . . . . .    
437	T T T T T T T T T T . . T T T . . . . . . .    
438	G G G G G G G G G G . . G G G . . . . . . .    
439	T T T T T T T T T T . . T T T . . . . . . .    
440	C C C C C C C C C C . . C C C . . . . . . .    
441	C C C C C C C C C C . . C C C . . . . . . .    
442	C C C C C C C C C C . . C C C . . . . . . .    
443	A A A A A A A A A A . . A A A . . . . . . .    
444	T T T T T T T T T T . . T T T . . . . . . .    
445	A A A A A A A A A A . . A A A . . . . . . .    
446	G G G G G G G G G G . . G G G . . . . . . .    
447	C C C C C C C C C C . . C C C . . . . . . .    
448	T T T T T T T T T T . . T T T . . . . . . .    
449	A A A A A A A A A A . . A A A . . . . . . .    
450	T T T T T T T T T T . . T T T . . . . . . .    
451	A A A A A A A A A A . . A A A . . . . . . .    
452	C C C C C C C C C C . . C C C . . . . . . .    
453	G G G G G G G G G G . . G G G . . . . . . .    
454	A A A A A A A A A A . . A A A . . . . . . .    
455	C C C C C C C C C C . . C C C . . . . . . .    
456	G G G G G G G G G G . . G G G . . . . . . .    
457	A A A A A A A A A A . . A A A . . . . . . .    
458	A A A A A A A A A A . . A A A . . . . . . .    
459	C C C C C C C C C C . . C C C . . . . . . .    
460	T C C C C C C C C C . . C C C . . . . . . .    
461	. A A A A A A A A A . . A A A A . . . . . .    
462	. A A A A A A A A A . . A A A A . . . . . .    
463	. T T T T T T T T T . . T T T T . . . . . .    
464	. G G G G G G G G G . . G G G G . . . . . .    
465	. T T T T T T T T T . . T T T T . . . . . .    
466	. T T T T T T T T T . . T T T T . . . . . .    
467	. T T T T T T T T T . . T T T T . . . . . .    
468	. A A A A A A A A A . . A A A A . . . . . .    
469	. C C C C C C C C C . . C C C C . . . . . .    
470	. C C C C C C C C C . . C C C C . . . . . .    
471	. A A A A A A A A A . . A A A A . . . A A .    
472	. T T T T T T T T T . . T T T T . . . T T .    
473	. C C C C C C C C C . . C C C C . . . C C .    
474	. T T T T T T T T T . . T T T T . . . T T .    
475	. G G G G G G G G G . . G G G G . . . G G .    
476	. C C C C C C C C C . . C C C C . . . C C .    
477	. T T T T T T T T T . . T T T T . . . T T .    
478	. C C C C C C C C C . . C C C C . . . C C .    
479	. A A A A A A A A A . . A A A A . . . A A .    
480	. A A A A A A A A A . . A A A A . . . A A .    
481	. A A A A A A A A A . . A A A A . . . A A .    
482	. C C C C C C C C C . . C C C C . . . C C .    
483	. T T T T T T T T T . . T T T T . . . T T .    
484	. T T T T T T T T T . . T T T T . . . T T .    
485	. C C C C C C C C C . . C C C C . . . C C .    
486	. C C C C C C C C C . . C C C C . . . C C .    
487	. A A A A A A A A A . . A A A A . . . A A .    
488	. G G G G G G G G G . . G G G G . . . G G .    
489	. C C C C C C C C C . . C C C C . . . C C .    
490	. C C C C C C C C C . . C C C C . . . C C .    
491	. G G G G G G G G G . . G G G G . . . G G .    
492	. T T T T T T T T T . . T T T T . . . T T .    
493	. C C C C C C C C C . . C C C C . . . C C .    
494	. T T T T T T T T T . . T T T T . . . T T .    
495	. G G G G G G G G G . . G G G G . . . G G .    
496	. A A A A A A A A A . . A A A A . . . A A .    
497	. C C C C C C C C C . . C C C C . . . C C .    
498	. A A A A A A A A A . . A A A A . . . A A .    
499	. T T T T T T T T T . . T T T T . . . T T .    
500	. G G G G G G G G G . . G G G G . . . G G .    
501	. G G G G G G G G G . . G G G G . . . G G .    
502	. T T T T T T T T T . . T T T T . . . T T .    
503	. G G G G G G G G G . . G G G G . . . G G .    
504	. G G G G G G G G G . . G G G G . . . G G .    
505	. A A A A A A A A A . . A A A A . . . A A .    
506	. A A A A A A A A A . . A A A A . . . A A .    
507	. A A A A A A A A A . . A A A A . . . A A .    
508	. C C C C C C C C C . . C C C C . . . C C .    
509	. C C C C C C C C C . . C C C C . . . C C .    
510	. A A A A A A A A A . . A A A A . . . A A .    
511	. T T T T T T T T T . . T T T T . . . T T .    
512	. C C C C C C C C C . . C C C C . . . C C .    
513	. T T T T T T T T T . . T T T T . . . T T .    
514	. G G G G G G G G G . . G G G G . . . G G .    
515	. G G G G G G G G G . . G G G G . . . G G .    
516	. G G G G G G G G G . . G G G G . . . G G .    
517	. T T T T T T T T T . . T T T T . . . T T .    
518	. C C G C C G G G C . . G G C C . . . G G .  7	 transversion  
519	. A A A A A A A A A . . A A A A A . . A A A    
520	. G G G G G G G G G . . G G G G G . . G G G    
521	. C C C C C C C C C . . C C C C C . . C C C    
522	. . T T T T T T T T . . T T T T T . . T T T    
523	. . C C C C C C C C . . C C C C C . . C C C    
524	. . C C C C C C C C . . C C C C C . . C C C    
525	. . T T T T T T T T . . T T T T T . . T T T    
526	. . C C C C C C C C . . C C C C C . . C C C    
527	. . C C C C C C C C . . C C C C C . . C C C    
528	. . T T T T T T T T . . T T T T T . . T T T    
529	. . C C C C C C C C . . C C C C C . . C C C    
530	. . C C C C C C C C . . C C C C C . . C C C    
531	. . T T T T T T T T . . T T T T T . . T T T    
532	. . G G G G G G G G . . G G G G G . . G G G    
533	. . G G G G G G G G . . G G G G G . . G G G    
534	. . C C C C C C C C . C C C C C C . . C C C    
535	. . A A A A A A A A . A A A A A A . . A A A    
536	. . C C C C C C C C . C C C C C C . . C C C    
537	. . T T T T T T T T . T T T T T T . . T T T    
538	. . G G G G G G G G . G G G G G G . . G G G    
539	. . C C C C C C C C . C C C C C C . . C C C    
540	. . A A A A A A A A . A A A A A A . . A A A    
541	. . G G G G G G G G . G G G G G G . . G G G    
542	. . C C C C C C C C . C C C C C C . . C C C    
543	. . A A A A A A A A . A A A A A A . . A A A    
544	. . C C C C C C C C . C C C C C C . . C C C    
545	. . C C C C C C C C . C C C C C C . . C C C    
546	. . C C C C C C C C . C C C C C C . . C C C    
547	. . G G G G G G G G . G G G G G G . . G G G    
548	. . G G G G G G G G . G G G G G G . . G G G    
549	. . T T T T T T T T . T T T T T T . . T T T    
550	. . A A A A A A A A . A A A A A A . . A A A    
551	. . C C C C C C C C . C C C C C C . . C C C    
552	. . A A A A A A A A . A A A A A A . . A A A    
553	. . C C C C C C C C . C C C C C C . . C C C    
554	. . A A A A A A A A . A A A A A A . . A A A    
555	. . T T T T T T T T . T T T T T T . . T T T    
556	. . G G G G G G G G . G G G G G G . . G G G    
557	. . T T T T T T T T . T T T T T T . . T T T    
558	. . T T T T T T T T . T T T T T T . . T T T    
559	. . T . T T T T T T . T T T T T T . . T T T    
560	. . A . A A A A A A . A A A A A A . . A A A    
561	. . G . G G G G G G . G G G G G G . . G G G    
562	. . G . G G G G G G . G G G G G G . . G G G    
563	. . A . A A A A A A . A A A A A A . . A A A    
564	. . G . G G G G G G . G G G G G G . . G G G    
565	. . G . G G G G G G . G G G G G G . . G G G    
566	. . C . C C C C C C . C C C C C C . . C C C    
567	. . A . A A A A A A . A A A A A A . . A A A    
568	. . C . C C C C C C . C C C C C C . . C C C    
569	. . C . C C C C C C . C C C C C C . . C C C    
570	. . T . T T T T T T . T T T T T T . . T T T    
571	. . G . G G G G G G . G G G G G G . . G G G    
572	. . C . C C C - - C . C - - C C - . . - C C  6	 indel  
573	. . T . T T T - - T . T - - T T - . . - T T  6	 indel  
574	. . G . G G G - - G . G - - G G - . . - G G  6	 indel  
575	. . T . T T T T T T . T T T T T T . . T T T    
576	. . G . G G G G G G . G G G G G G . . G G G    
577	. . T . T T T T T T . T T T T T T . . T T T    
578	. . G . G G G G G G . G G G G G G . . G G G    
579	. . A . A A A A A A . A A A A A A . . A A A    
580	. . . . C C C C C C . C C C C C C . . C C C    
581	. . . . T T T T T T . T T T T T T . . T T T    
582	. . . . T T T T T T . T T T T T T . . T T T    
583	. . . . T T T T T T . C T T T T T . . T T T    
584	. . . . A A A A A A . A A A A A A . . A A A    
585	. . . . C C C C C C . C C C C C C . . C C C    
586	. . . . A A A A A A . A A A A A A . . A A A    
587	. . . . G G G G G G . G G G G G G . . G G G    
588	. . . . T T T T T T . T T T T T T . . T T T    
589	. . . . C C C C C C . C C C C C C . . C C C    
590	. . . . C C C C C C . C C C C C C . . C C C    
591	. . . . C C C C C C . C C C C C C . . C C C    
592	. . . . G G G G G G . G G G G G G . . G G G    
593	. . . . C C C C C C . C C C C C C . . C C C    
594	. . . . T T T T T T . T T T T T T . . T T T    
595	. . . . C C C C C C . C C C C C C . . C C C    
596	. . . . C C C C C C . C C C C C C . . C C C    
597	. . . . A A A A A A . A A A A A A . . A A A    
598	. . . . G G G G G G . G G G G G G . . G G G    
599	. . . . A A A A A A . A A A A A A . . A A A    
600	. . . . A A A A A A . A A A A A A . . A A A    
601	. . . . T T T T T T . T T T T T T . . T T T    
602	. . . . C C C C C C . C C C C C C . . C C C    
603	. . . . C C C C C C . C C C C C C . . C C C    
604	. . . . T T T T T T . T T T T T T . . T T T    
605	. . . . C C C C C C . C C C C C C . . C C C    
606	. . . . T T T T T T . T T T T T T . . T T T    
607	. . . . C C C C C C . C C C C C C . . C C C    
608	. . . . C C C C C C . C C C C C C . . C C C    
609	. . . . T T T T T T . T T T T T T . . T T T    
610	. . . . T T T T T T . T T T T T T . . T T T    
611	. . . . G G G G G G . G G G G G G . . G G G    
612	. . . . A A A A A A . A A A A A A . . A A A    
613	. . . . T T T T T T . T T T T T T . . T T T    
614	. . . . C C C C C C . C C C C C C . . C C C    
615	. . . . A A A A A A . A A A A A A . . A A A    
616	. . . . G G G G G G . G G G G G G . . G G G    
617	. . . . T T T T T T . T T T T T T . . T T T    
618	. . . . T T T T T T . T T T T T T . . T T T    
619	. . . . T T T T T T . T T T T T T . . T T T    
620	. . . . T T T T T T . T T T T T T . . T T T    
621	. . . . A A A A A A . A A A A A A . . A A A    
622	. . . . T T T T T T . T T T T T T . . T T T    
623	. . . . C C C C C C . C C C C C C . . C C C    
624	. . . . A A A A A A . A A A A A A . . A A A    
625	. . . . G G G G G G . G G G G G G . . G G G    
626	. . . . A A A A A A . A A A A A A . . A A A    
627	. . . . G G G G G G . G G G G G G . . G G G    
628	. . . . G G G G G G . G G G G G G . . G G G    
629	. . . . A A A A A A . A A A A A A . . A A A    
630	. . . . . G G G G G . G G G G G G . . G G G    
631	. . . . . T T T T T . T T T T T T . . T T T    
632	. . . . . G G G G G . G G G G G G . . G G G    
633	. . . . . A A A A A . A A A A A A . A A A A    
634	. . . . . T T T T T . T T T T T T . T T T T    
635	. . . . . G G G G G . G G G G G G . G G G G    
636	. . . . . T T T T T . T T T T T T . T T T T    
637	. . . . . T T T T T . T T T T T T . T T T T    
638	. . . . . G G G G G G G G G G G G . G G G G    
639	. . . . . T T T T T T T T T T T T . T T T T    
640	. . . . . A A A A A A A A A A A A . A A A A    
641	. . . . . G G G G G G G G G G G G . G G G G    
642	. . . . . A A A A A A A A A A A A . A A A A    
643	. . . . . A A A A A A A A A A A A . A A A A    
644	. . . . . C C C C C C C C C C C C . C C C C    
645	. . . . . T T T T T T T T T T T T . T T T T    
646	. . . . . C C C C C C C C C C C C . C C C C    
647	. . . . . A A A A A A A A A A A A . A A A A    
648	. . . . . G G G G G G G G G G G G . G G G G    
649	. . . . . C C C C C C C C C C C C . C C C C    
650	. . . . . T T T T T T T T T T T T . T T T T    
651	. . . . . T T T T T T T T T T T T . T T T T    
652	. . . . . C C C C C C C C C C C C . C C C C    
653	. . . . . C C C C C C C C C C C C . C C C C    
654	. . . . . T T T T T T T T T T T T . T T T T    
655	. . . . . C C C C C C C C C C C C . C C C C    
656	. . . . . T T T T T T T T T T T T . T T T T    
657	. . . . . A A A A A A A A A A A A . A A A A    
658	. . . . . A A A A A A A A A A A A . A A A A    
659	. . . . . C C C C C C C C C C C C . C C C C    
660	. . . . . A A A A A A A A A A A A . A A A A    
661	. . . . . C C C C C C C C C C C C . C C C C    
662	. . . . . A A A A A A A A A A A A . A A A A    
663	. . . . . C C C C C C C C C C C C . C C C C    
664	. . . . . C C C C C C C C C C C C . C C C C    
665	. . . . . T T T T T T T T T T T T . T T T T    
666	. . . . . T T T T T T T T T T T T . T T T T    
667	. . . . . C C C C C C C C C C C C . C C C C    
668	. . . . . T T T T T T T T T T T T . T T T T    
669	. . . . . T T T T T T T T T T T T . T T T T    
670	. . . . . C C C C C C C C C C C C . C C C C    
671	. . . . . A A A A A A A A A A A A . A A A A    
672	. . . . . G G G G G G G G G G G G . G G G G    
673	. . . . . C C C C C C C C C C C C . C C C C    
674	. . . . . C C C C C C C C C C C C . C C C C    
675	. . . . . A A A A A A A A A A A A . A A A A    
676	. . . . . G G G G G G G G G G G G . G G G G    
677	. . . . . C C C C C C C C C C C C . C C C C    
678	. . . . . T T T T T T T T T T T T . T T T T    
679	. . . . . C C C C C C C C C C C C . C C C C    
680	. . . . . C C C C C C C C C C C C . C C C C    
681	. . . . . T T T T T T T T T T T T . T T T T    
682	. . . . . T T T T T T T T T T T T . T T T T    
683	. . . . . G G G G G G G G G G G G . G G G G    
684	. . . . . T T T T T T T T T T T T . T T T T    
685	. . . . . T T T T T T T T T T T T . T T T T    
686	. . . . . G G G G G G G G G G G G . G G G G    
687	. . . . . T T T T T T T T T T T T . T T T T    
688	. . . . . A . A A A A A A A A A A . A A A A    
689	. . . . . G . G G G G G G G G G G . G G G G    
690	. . . . . A . A A A A A A A A A A . A A A A    
691	. . . . . C . C C C T C C C C C C . C C C C    
692	. . . . . C . C C C C C C C C C C . C C C C    
693	. . . . . A . A A A A A A A A A A . A A A A    
694	. . . . . G . G G G G G G G G G G . G G G G    
695	. . . . . T . T T T T T T T T T T . T T T T    
696	. . . . . T . T T T T T T T T T T . T T T T    
697	. . . . . T . T T T T T T T T T T . T T T T    
698	. . . . . G . G G G G G G G G G G . G G G G    
699	. . . . . C . C C C C C C C C C C . C C C C    
700	. . . . . C . C C C C C C C C C C . C C C C    
701	. . . . . G . G G G G G G G G G G . G G G G    
702	. . . . . T . T T T G T T T T T T . T T T T    
703	. . . . . G . G G G G G G G G G G . G G G G    
704	. . . . . T . T T T T T T T T T T . T T T T    
705	. . . . . C . C C C C C C C C C C . C C C C    
706	. . . . . G . G G G G G G G G G G . G G G G    
707	. . . . . C . C C C C C C C C C C . C C C C    
708	. . . . . A . A A A A A A A A A A . A A A A    
709	. . . . . G . G G G G G G G G G G . G G G G    
710	. . . . . G . G G G G G G G G G G G G G G G    
711	. . . . . T . T T T T T T T T T T T T T T T    
712	. . . . . A . A A A A A A A A A A A A A A A    
713	. . . . . G . G G G G G G G G G G G G G G G    
714	. . . . . T . T T T T T T T T T T T T T T T    
715	. . . . . T . T T T T T T T T T T T T T T T    
716	. . . . . T . A A A A A A A A A A A A A A A    
717	. . . . . . . A A A A A A A A A A A A A A A    
718	. . . . . . . G G G G G G G G G G G G G G G    
719	. . . . . . . G G G G G G G G G G G G G G G    
720	. . . . . . . A A A A A A A A A A A A A A A    
721	. . . . . . . T T T T T T T T T T T T T T T    
722	. . . . . . . G G G G G G G G G G G G G G G    
723	. . . . . . . G G G G G G G G G G G G G G G    
724	. . . . . . . G G G G G G G G G G G G G G G    
725	. . . . . . . G G G G G G G G G G G G G G G    
726	. . . . . . . C C C C C C C C C C C C C C C    
727	. . . . . . . C C C A C C C C C C C C C C C    
728	. . . . . . . G G G G G G G G G G G G G G G    
729	. . . . . . . A A A A A A A A A A A A A A A    
730	. . . . . . . A A A A A A A A A A A A A A A    
731	. . . . . . . G G G G G G G G G G G G G G G    
732	. . . . . . . T T T T T T T T T T T T T T T    
733	. . . . . . . A A A A A A A A A A A A A A A    
734	. . . . . . . G G G G G G G G G G G G G G G    
735	. . . . . . . G G G G G G G G G G G G G G G    
736	. . . . . . . T T T T T T T T T T T T T T T    
737	. . . . . . . G G G G G G G G G G G G G G G    
738	. . . . . . . G G G G G G G G G G G G G G G    
739	. . . . . . . G G G G G G G G G G G G G G G    
740	. . . . . . . G G G G G G G G G G G G G G G    
741	. . . . . . . T T T T T T T T T T T T T T T    
742	. . . . . . . C C C C C C C C C C C C C C C    
743	. . . . . . . T T T T T T T T T T T T T T T    
744	. . . . . . . C C C C C C C C C C C C C C C    
745	. . . . . . . T T T T T T T T T T T T T T T    
746	. . . . . . . G G G G G G G G G G G G G G G    
747	. . . . . . . T T T T T T T T T T T T T T T    
748	. . . . . . . C C C C C C C C C C C C C C C    
749	. . . . . . . T . T T T T T T T T T T T T T    
750	. . . . . . . A . A A A A A A A A A A A A A    
751	. . . . . . . T . T T T T T T T T T T T T T    
752	. . . . . . . C . C C C C C C C C C C C C C    
753	. . . . . . . A . A A A A A A A A A A A A A    
754	. . . . . . . C . C C C C C C C C C C C C C    
755	. . . . . . . A . A A A A A A A A A A A A A    
756	. . . . . . . T . T T T T T T T T T T T T T    
757	. . . . . . . A . A A A A A A A A A A A A A    
758	. . . . . . . G . G G G G G G G G G G G G G    
759	. . . . . . . G . G G G G G G G G G G G G G    
760	. . . . . . . C . C C C C C C C C C C C C C    
761	. . . . . . . C . C C C C C C C C C C C C C    
762	. . . . . . . C . C C C C C C C C C C C C C    
763	. . . . . . . C . C C C C C C C C C C C C C    
764	. . . . . . . T . T T T T T T T T T T T T T    
765	. . . . . . . G . G G G G G G G G G G G G G    
766	. . . . . . . T . T T T T T T T T T T T T T    
767	. . . . . . . C . C C C C C C C C C C C C C    
768	. . . . . . . T . T T T T T T T T T T T T T    
769	. . . . . . . C . C C C C C C C C C C C C C    
770	. . . . . . . G . G G G G G G G G G G G G G    
771	. . . . . . . T . T T T T T T T T T T T T T    
772	. . . . . . . . . C C C C C C C C C C C C C    
773	. . . . . . . . . C C C C C C C C C C C C C    
774	. . . . . . . . . G G G G G G G G G G G G G    
775	. . . . . . . . . T T T T T T T T T T T T T    
776	. . . . . . . . . G G G G G G G G G G G G G    
777	. . . . . . . . . T T T T T T T T T T T T T    
778	. . . . . . . . . C C C C C C C C C C C C C    
779	. . . . . . . . . C C C C C C C C C C C C C    
780	. . . . . . . . . G G G G G G G G G G G G G    
781	. . . . . . . . . A A A A A A A A A A A A A    
782	. . . . . . . . . A A A A A A A A A A A A A    
783	. . . . . . . . . T T T T T T T T T T T T T    
784	. . . . . . . . . G G G G G G G G G G G G G    
785	. . . . . . . . . T T T T T T T T T T T T T    
786	. . . . . . . . . A A A A A A A A A A A A A    
787	. . . . . . . . . A A A A A A A A A A A A A    
788	. . . . . . . . . . G G G G G G G G G G G G    
789	. . . . . . . . . . T T T T T T T T T T T T    
790	. . . . . . . . . . C C C C C C C C C C C C    
791	. . . . . . . . . . C C C C C C C C C C C C    
792	. . . . . . . . . . T T T T T T T T T T T T    
793	. . . . . . . . . . G G G G G G G G G G G G    
794	. . . . . . . . . . T T T T T T T T T T T T    
795	. . . . . . . . . . T T T T T T T T T T T T    
796	. . . . . . . . . . G G G G G G G G G G G G    
797	. . . . . . . . . . T T T T T T T T T T T T    
798	. . . . . . . . . . T T T T T T T T T T T T    
799	. . . . . . . . . . G G G G G G G G G G G G    
800	. . . . . . . . . . A A A A A A A A A A A A    
801	. . . . . . . . . . C C C C C C C C C C C C    
802	. . . . . . . . . . A A A A A A A A A A A A    
803	. . . . . . . . . . G G G G G G G G G G G G    
804	. . . . . . . . . . A A A A A A A A A A A A    
805	. . . . . . . . . . G G G G G G G G G G G G    
806	. . . . . . . . . . C C C C C C C C C C C C    
807	. . . . . . . . . . C C C C C C C C C C C C    
808	. . . . . . . . . . G G G G G G G G G G G G    
809	. . . . . . . . . . A A A A A A A A A A A A    
810	. . . . . . . . . . T T T T T T T T T T T T    
811	. . . . . . . . . . A A A A A A A A A A A A    
812	. . . . . . . . . . G G G G G G G G G G G G    
813	. . . . . . . . . . A A A A A A A A A A A A    
814	. . . . . . . . . . G G G G G G G G G G G G    
815	. . . . . . . . . . A A A A A A A A A A A A    
816	. . . . . . . . . . A A A A A A A A A A A A    
817	. . . . . . . . . . A A A A A A A A A A A A    
818	. . . . . . . . . . G G G G G G G G G G G G    
819	. . . . . . . . . . G G G G G G G G G G G G    
820	. . . . . . . . . . A A A A A A A A A A A A    
821	. . . . . . . . . . C C C C C C C C C C C C    
822	. . . . . . . . . . G G G G G G G G G G G G    
823	. . . . . . . . . . T T T T T T T T T T T T    
824	. . . . . . . . . . T T T T T T T T T T T T    
825	. . . . . . . . . . T T T T T T T T T T T T    
826	. . . . . . . . . . G G G G G G G G G G G G    
827	. . . . . . . . . . T T T T T T T T T T T T    
828	. . . . . . . . . . T T T T T T T T T T T T    
829	. . . . . . . . . . C C C C C C C C C C C C    
830	. . . . . . . . . . T T T T T T T T T T T T    
831	. . . . . . . . . . T T T T T T T T T T T T    
832	. . . . . . . . . . T T T T T T T T T T T T    
833	. . . . . . . . . . G G G G G G G G G G G G    
834	. . . . . . . . . . A A A A A A A A A A A A    
835	. . . . . . . . . . G G G G G G G G G G G G    
836	. . . . . . . . . . T T T T T T T T T T T T    
837	. . . . . . . . . . A A A A A A A A A A A A    
838	. . . . . . . . . . G G G G G G G G G G G G    
839	. . . . . . . . . . G G G G G G G G G G G G    
840	. . . . . . . . . . G G G G G G G G G G G G    
841	. . . . . . . . . . T T T T T T T T T T T T    
842	. . . . . . . . . . A A A A A A A A A A A A    
843	. . . . . . . . . . T T T T T T T T T T T T    
844	. . . . . . . . . . G G G G G G G G G G G G    
845	. . . . . . . . . . C C C C C C C C C C C C    
846	. . . . . . . . . . C C C C C C C C C C C C    
847	. . . . . . . . . . G G G G G G G G G G G G    
848	. . . . . . . . . . C C C C C C C C C C C C    
849	. . . . . . . . . . G G G G G G G G G G G G    
850	. . . . . . . . . . T T T T T T T T T T T T    
851	. . . . . . . . . . T T T T T T T T T T T T    
852	. . . . . . . . . . G G G G G G G G G G G G    
853	. . . . . . . . . . T T T T T T T T T T T T    
854	. . . . . . . . . . G G G G G G G G G G G G    
855	. . . . . . . . . . A A A A A A A A A A A A    
856	. . . . . . . . . . G G G G G G G G G G G G    
857	. . . . . . . . . . G G G G G G G G G G G G    
858	. . . . . . . . . . A A A A A A A A A A A A    
859	. . . . . . . . . . A A A A A A A A A A A A    
860	. . . . . . . . . . T T T T T T T T T T T T    
861	. . . . . . . . . . A A A A A A A A A A A A    
862	. . . . . . . . . . C C C C C C C C C C C C    
863	. . . . . . . . . . T T T T T T T T T T T T    
864	. . . . . . . . . . G G G G G G G G G G G G    
865	. . . . . . . . . . T T T T T T T T T T T T    
866	. . . . . . . . . . G G G G G G G G G G G G    
867	. . . . . . . . . . C C C C C C C C C C C C    
868	. . . . . . . . . . C C C C C C C C C C C C    
869	. . . . . . . . . . G G G G G G G G G G G G    
870	. . . . . . . . . . C C C C C C C C C C C C    
871	. . . . . . . . . . C C C C C C C C C C C C    
872	. . . . . . . . . . G G G G G G G G G G G G    
873	. . . . . . . . . . A A A A A A A A A A A A    
874	. . . . . . . . . . C C C C C C C C C C C C    
875	. . . . . . . . . . G G G G G G G G G G G G    
876	. . . . . . . . . . T T T T T T T T T T T T    
877	. . . . . . . . . . T T T T T T T T T T T T    
878	. . . . . . . . . . C C C C C C C C C C C C    
879	. . . . . . . . . . A A A A A A A A A A A A    
880	. . . . . . . . . . A A A A A A A A A A A A    
881	. . . . . . . . . . C C C C C C C C C C C C    
882	. . . . . . . . . . C C C C C C C C C C C C    
883	. . . . . . . . . . G G G G G G G G G G G G    
884	. . . . . . . . . . G G G G G G G G G G G G    
885	. . . . . . . . . . A A A A A A A A A A A A    
886	. . . . . . . . . . C C C C C C C C C C C C    
887	. . . . . . . . . . C C C C C C C C C C C C    
888	. . . . . . . . . . C C C C C C C C C C C C    
889	. . . . . . . . . . A A A A A A A A A A A A    
890	. . . . . . . . . . T T T T T T T T T T T T    
891	. . . . . . . . . . T T T T T T T T T T T T    
892	. . . . . . . . . . T T T T T T T T T T T T    
893	. . . . . . . . . . C C C C C C C C C C C C    
894	. . . . . . . . . . C C C C C C C C C C C C    
895	. . . . . . . . . . C C C C C C C C C C C C    
896	. . . . . . . . . . G G G G G G G G G G G G    
897	. . . . . . . . . . T T T T T T T T T T T T    
898	. . . . . . . . . . T T T T T T T T T T T T    
899	. . . . . . . . . . A A A A A A A A A A A A    
900	. . . . . . . . . . T T T T T T T T T T T T    
901	. . . . . . . . . . T T T T T T T T T T T T    
902	. . . . . . . . . . T T T T T T T T T T T T    
903	. . . . . . . . . . C C C C C C C C C C C C    
904	. . . . . . . . . . C C C C C C C C C C C C    
905	. . . . . . . . . . C C C C C C C C C C C C    
906	. . . . . . . . . . A A A A A A A A A A A A    
907	. . . . . . . . . . A A A A A A A A A A A A    
908	. . . . . . . . . . T T T T T T T T T T T T    
909	. . . . . . . . . . G G G G G G G G G G G G    
910	. . . . . . . . . . T T T T T T T T T T T T    
911	. . . . . . . . . . A A A A A A A A A A A A    
912	. . . . . . . . . . G G G G G G G G G G G G    
913	. . . . . . . . . . G G G G G G G G G G G G    
914	. . . . . . . . . . G G G G G G G G G G G G    
915	. . . . . . . . . . T T T T T T T T T T T T    
916	. . . . . . . . . . T T T T T T T T T T T T    
917	. . . . . . . . . . G G G G G G G G G G G G    
918	. . . . . . . . . . A A A A A A A A A A A A    
919	. . . . . . . . . . C C C C C C C C C C C C    
920	. . . . . . . . . . T T T T T T T T T T T T    
921	. . . . . . . . . . C C C C C C C C C C C C    
922	. . . . . . . . . . C C C C C C C C C C C C    
923	. . . . . . . . . . A A A A A A A A A A A A    
924	. . . . . . . . . . G G G G G G G G G G G G    
925	. . . . . . . . . . A A A A A A A A A A A A    
926	. . . . . . . . . . G G G G G G G G G G G G    
927	. . . . . . . . . . C C C C C C C C C C C C    
928	. . . . . . . . . . C C C C C C C C C C C C    
929	. . . . . . . . . . G G G G G G G G G G G G    
930	. . . . . . . . . . T T T T T T T T T T T T    
931	. . . . . . . . . . T T T T T T T T T T T T    
932	. . . . . . . . . . C C C C C C C C C C C C    
933	. . . . . . . . . . C C C C C C C C C C C C    
934	. . . . . . . . . . C C C C C C C C C C C C    
935	. . . . . . . . . . G G G G G G G G G G G G    
936	. . . . . . . . . . A A A A A A A A A A A A    
937	. . . . . . . . . . T T T T T T T T T T T T    
938	. . . . . . . . . . G G G G G G G G G G G G    
939	. . . . . . . . . . C C C C C C C C C C C C    
940	. . . . . . . . . . T T T T T T T T T T T T    
941	. . . . . . . . . . G G G G G G G G G G G G    
942	. . . . . . . . . . T T T T T T T T T T T T    
943	. . . . . . . . . . G G G G G G G G G G G G    
944	. . . . . . . . . . A A A A A A A A A A A A    
945	. . . . . . . . . . G G G G G G G G G G G G    
946	. . . . . . . . . . T T T T T T T T T T T T    
947	. . . . . . . . . . T T T T T T T T T T T T    
948	. . . . . . . . . . C C C C C C C C C C C C    
949	. . . . . . . . . . C C C C C C C C C C C C    
950	. . . . . . . . . . T T T T T T T T T T T T    
951	. . . . . . . . . . G G G G G G G G G G G G    
952	. . . . . . . . . . T T T T T T T T T T T T    
953	. . . . . . . . . . C C C C C C C C C C C C    
954	. . . . . . . . . . C C C C C C C C C C C C    
955	. . . . . . . . . . C C C C C C C C C C C C    
956	. . . . . . . . . . G G G G G G G G G G G G    
957	. . . . . . . . . . C C C C C C C C C C C C    
958	. . . . . . . . . . T T T T T T T T T T T T    
959	. . . . . . . . . . C C C C C C C C C C C C    
960	. . . . . . . . . . G G G G G G G G G G G G    
961	. . . . . . . . . . A A A A A A A A A A A A    
962	. . . . . . . . . . A A A A A A A A A A A A    
963	. . . . . . . . . . G G G G G G G G G G G G    
964	. . . . . . . . . . T T T T T T T T T T T T    
965	. . . . . . . . . . G G G G G G G G G G G G    
966	. . . . . . . . . . G G G G G G G G G G G G    
967	. . . . . . . . . . C C C C C C C C C C C C    
968	. . . . . . . . . . C C C C C C C C C C C C    
969	. . . . . . . . . . G G G G G G G G G G G G    
970	. . . . . . . . . . T T T T T T T T T T T T    
971	. . . . . . . . . . G G G G G G G G G G G G    
972	. . . . . . . . . . G G G G G G G G G G G G    
973	. . . . . . . . . . T T T T T T T T T T T T    
974	. . . . . . . . . . C C C C C C C C C C C C    
975	. . . . . . . . . . G G G G G G G G G G G G    
976	. . . . . . . . . . T T T T T T T T T T T T    
977	. . . . . . . . . . A A A A A A A A A A A A    
978	. . . . . . . . . . T T T T T T T T T T T T    
979	. . . . . . . . . . T T T T T T T T T T T T    
980	. . . . . . . . . . C C C C C C C C C C C C    
981	. . . . . . . . . . T T T T T T T T T T T T    
982	. . . . . . . . . . C C C C C C C C C C C C    
983	. . . . . . . . . . G G G G G G G G G G G G    
984	. . . . . . . . . . C C C C C C C C C C C C    
985	. . . . . . . . . . T T T T T T T T T T T T    
986	. . . . . . . . . . T T T T T T T T T T T T    
987	. . . . . . . . . . C C C C C C C C C C C C    
988	. . . . . . . . . . C C C C C C C C C C C C    
989	. . . . . . . . . . C C C C C C C C C C C C    
990	. . . . . . . . . . T T T T T T T T T T T T    
991	. . . . . . . . . . C C C C C C C C C C C C    
992	. . . . . . . . . . G G G G G G G G G G G G    
993	. . . . . . . . . . C C C C C C C C C C C C    
994	. . . . . . . . . . T T T T T T T T T T T T    
995	. . . . . . . . . . A A A A A A A A A A A A    
996	. . . . . . . . . . T T T T T T T T T T T T    
997	. . . . . . . . . . C C C C C C C C C C C C    
998	. . . . . . . . . . T T T T T T T T T T T T    
999	. . . . . . . . . . T T T T T T T T T T T T    
1000	. . . . . . . . . . T T T T T T T T T T T T    
1001	. . . . . . . . . . A A A A A A A A A A A A    
1002	. . . . . . . . . . T T T T T T T T T T T T    
1003	. . . . . . . . . . T T T T T T T T T T T T    
1004	. . . . . . . . . . A A A A A A A A A A A A    
1005	. . . . . . . . . . T T T T T T T T T T T T    
1006	. . . . . . . . . . T T T T T T T T T T T T    
1007	. . . . . . . . . . A A A A A A A A A A A A    
1008	. . . . . . . . . . T T T T T T T T T T T T    
1009	. . . . . . . . . . T T T T T T T T T T T T    
1010	. . . . . . . . . . G G G G G G G G G G G G    
1011	. . . . . . . . . . T T T T T T T T T T T T    
1012	. . . . . . . . . . T T T T T T T T T T T T    
1013	. . . . . . . . . . G G G G G G G G G G G G    
1014	. . . . . . . . . . T T T T T T T T T T T T    
1015	. . . . . . . . . . T T T T T T T T T T T T    
1016	. . . . . . . . . . G G G G G G G G G G G G    
1017	. . . . . . . . . . T T T T T T T T T T T T    
1018	. . . . . . . . . . T T T T T T T T T T T T    
1019	. . . . . . . . . . G G G G G G G G G G G G    
1020	. . . . . . . . . . T T T T T T T T T T T T    
1021	. . . . . . . . . . T T T T T T T T T T T T    
1022	. . . . . . . . . . A A A A A A A A A A A A    
1023	. . . . . . . . . . T T T T T T T T T T T T    
1024	. . . . . . . . . . T T T T T T T T T T T T    
1025	. . . . . . . . . . A A A A A A A A A A A A    
1026	. . . . . . . . . . T T T T T T T T T T T T    
1027	. . . . . . . . . . T T T T T T T T T T T T    
1028	. . . . . . . . . . G G G G G G G G G G G G    
1029	. . . . . . . . . . T T T T T T T T T T T T    
1030	. . . . . . . . . . T T T T T T T T T T T T    
1031	. . . . . . . . . . A A A A A A A A A A A A    
1032	. . . . . . . . . . T T T T T T T T T T T T    
1033	. . . . . . . . . . T T T T T T T T T T T T    
1034	. . . . . . . . . . G G G G G G G G G G G G    
1035	. . . . . . . . . . T T T T T T T T T T T T    
1036	. . . . . . . . . . A A A A A A A A A A A A    
1037	. . . . . . . . . . G G G G G G G G G G G G    
1038	. . . . . . . . . . C C C C C C C C C C C C    
1039	. . . . . . . . . . C C C C C C C C C C C C    
1040	. . . . . . . . . . A A A A A A A A A A A A    
1041	. . . . . . . . . . T T T T T T T T T T T T    
1042	. . . . . . . . . . G G G G G G G G G G G G    
1043	. . . . . . . . . . A A A A A A A A A A A A    
1044	. . . . . . . . . . G G G G G G G G G G G G    
1045	. . . . . . . . . . G G G G G G G G G G G G    
1046	. . . . . . . . . . T T T T T T T T T T T T    
1047	. . . . . . . . . . G G G G G G G G G G G G    
1048	. . . . . . . . . . T T T T T T T T T T T T    
1049	. . . . . . . . . . C C C C C C C C C C C C    
1050	. . . . . . . . . . G G G G G G G G G G G G    
1051	. . . . . . . . . . G G G G G G G G G G G G    
1052	. . . . . . . . . . T T T T T T T T T T T T    
1053	. . . . . . . . . . A A A A A A A A A A A A    
1054	. . . . . . . . . . A A A A A A A A A A A A    
1055	. . . . . . . . . . C C C C C C C C C C C C    
1056	. . . . . . . . . . G G G G G G G G G G G G    
1057	. . . . . . . . . . A A A A A A A A A A A A    
1058	. . . . . . . . . . A A A A A A A A A A A A    
1059	. . . . . . . . . . C C C C C C C C C C C C    
1060	. . . . . . . . . . C C C C C C C C C C C C    
1061	. . . . . . . . . . G G G G G G G G G G G G    
1062	. . . . . . . . . . A A A A A A A A A A A A    
1063	. . . . . . . . . . C C C C C C C C C C C C    
1064	. . . . . . . . . . T T T T T T T T T T T T    
1065	. . . . . . . . . . T T T T T T T T T T T T    
1066	. . . . . . . . . . C C C C C C C C C C C C    
1067	. . . . . . . . . . T T T T T T T T T T T T    
1068	. . . . . . . . . . G G G G G G G G G G G G    
1069	. . . . . . . . . . A A A A A A A A A A A A    
1070	. . . . . . . . . . T T T T T T T T T T T T    
1071	. . . . . . . . . . T T T T T T T T T T T T    
1072	. . . . . . . . . . C C C C C C C C C C C C    
1073	. . . . . . . . . . A A A A A A A A A A A A    
1074	. . . . . . . . . . G G G G G G G G G G G G    
1075	. . . . . . . . . . C C C C C C C C C C C C    
1076	. . . . . . . . . . C C C C C C C C C C C C    
1077	. . . . . . . . . . T T T T T T T T T T T T    
1078	. . . . . . . . . . G G G G G G G G G G G G    
1079	. . . . . . . . . . C C C C C C C C C C C C    
1080	. . . . . . . . . . G G G G G G G G G G G G    
1081	. . . . . . . . . . A A A A A A A A A A A A    
1082	. . . . . . . . . . C C C C C C C C C C C C    
1083	. . . . . . . . . . A A A A A A A A A A A A    
1084	. . . . . . . . . . C C C C C C C C C C C C    
1085	. . . . . . . . . . A A A A A A A A A A A A    
1086	. . . . . . . . . . G G G G G G G G G G G G    
1087	. . . . . . . . . . A A A A A A A A A A A A    
1088	. . . . . . . . . . T T T T T T T T T T T T    
1089	. . . . . . . . . . T T T T T T T T T T T T    
1090	. . . . . . . . . . T T T T T T T T T T T T    
1091	. . . . . . . . . . C C C C C C C C C C C C    
1092	. . . . . . . . . . C C C C C C C C C C C C    
1093	. . . . . . . . . . T T T T T T T T T T T T    
1094	. . . . . . . . . . T T T T T T T T T T T T    
1095	. . . . . . . . . . T T T T T T T T T T T T    
1096	. . . . . . . . . . T T T T T T T T T T T T    
1097	. . . . . . . . . . C C C C C C C C C C C C    
1098	. . . . . . . . . . T T T T T T T T T T T T    
1099	. . . . . . . . . . C C C C C C C C C C C C    
1100	. . . . . . . . . . C C C C C C C C C C C C    
1101	. . . . . . . . . . T T T T T T T T T T T T    
1102	. . . . . . . . . . C C C C C C C C C C C C    
1103	. . . . . . . . . . C C C C C C C C C C C C    
1104	. . . . . . . . . . G G G G G G G G G G G G    
1105	. . . . . . . . . . T T T T T T T T T C T T    
1106	. . . . . . . . . . T T T T T T T T T T T T    
1107	. . . . . . . . . . G G G G G G G G G G G G    
1108	. . . . . . . . . . C C C C C C C C C C C C    
1109	. . . . . . . . . . C C C C C C C C C C C C    
1110	. . . . . . . . . . A A A A A A A A A A A A    
1111	. . . . . . . . . . T T T T T T T T T T T T    
1112	. . . . . . . . . . T T T T T T T T T T T T    
1113	. . . . . . . . . . A A A A A A A A A A A A    
1114	. . . . . . . . . . G G G G G G G G G G G G    
1115	. . . . . . . . . . G G G G G G G G G G G G    
1116	. . . . . . . . . . A A A A A A A A A A A A    
1117	. . . . . . . . . . A A A A A A A A A A A A    
1118	. . . . . . . . . . A A A A A A A A A A A A    
1119	. . . . . . . . . . T T T T T T T T T T T T    
1120	. . . . . . . . . . A A A A A A A A A A A A    
1121	. . . . . . . . . . G G G G G G G G G G G G    
1122	. . . . . . . . . . C C C C C C C C C C C C    
1123	. . . . . . . . . . A A A A A A A A A A A A    
1124	. . . . . . . . . . A A A A A A A A A A A A    
1125	. . . . . . . . . . T T T T T T T T T T T T    
1126	. . . . . . . . . . G . G G G G G G G G G G    
1127	. . . . . . . . . . G . G G G G G G G G G G    
1128	. . . . . . . . . . A . A A A A A A A A A A    
1129	. . . . . . . . . . T . T T T T T T T T T T    
1130	. . . . . . . . . . G . G G G G G G G G G G    
1131	. . . . . . . . . . C . C C C C C C C C C C    
1132	. . . . . . . . . . A . A A A A A A A A A A    
1133	. . . . . . . . . . . . A A A A A A A A A A    
1134	. . . . . . . . . . . . G G G G G G G G G G    
1135	. . . . . . . . . . . . C C C C C C C C C C    
1136	. . . . . . . . . . . . C A A A A A A A A A    
1137	. . . . . . . . . . . . . A A A A A A A A A    
1138	. . . . . . . . . . . . . C C C C C C C C C    
1139	. . . . . . . . . . . . . G G G G G G G G G    
1140	. . . . . . . . . . . . . T T T T T T T T T    
1141	. . . . . . . . . . . . . T T T T T T T T T    
1142	. . . . . . . . . . . . . A A A A A A A A A    
1143	. . . . . . . . . . . . . A A A A A A A A A    
1144	. . . . . . . . . . . . . A A A A A A A A A    
1145	. . . . . . . . . . . . . A A A A A A A A A    
1146	. . . . . . . . . . . . . A A A A A A A A A    
1147	. . . . . . . . . . . . . G G G G G G G G G    
1148	. . . . . . . . . . . . . C C C C C C C C C    
1149	. . . . . . . . . . . . . A A A A A A A A A    
1150	. . . . . . . . . . . . . A C A A A A A A A    
1151	. . . . . . . . . . . . . T . T T T T T T T    
1152	. . . . . . . . . . . . . A . A A A A A A A    
1153	. . . . . . . . . . . . . G . G G G G G G G    
1154	. . . . . . . . . . . . . C . C C C C C C C    
1155	. . . . . . . . . . . . . T . T T T T T T T    
1156	. . . . . . . . . . . . . T . T T T T T T T    
1157	. . . . . . . . . . . . . C . G G G G G G G    
1158	. . . . . . . . . . . . . . . C C C C C C C    
1159	. . . . . . . . . . . . . . . G G G G G G G    
1160	. . . . . . . . . . . . . . . T T T T T T T    
1161	. . . . . . . . . . . . . . . A A A A A A A    
1162	. . . . . . . . . . . . . . . C C C C C C C    
1163	. . . . . . . . . . . . . . . C C C C C C C    
1164	. . . . . . . . . . . . . . . A A A A A A A    
1165	. . . . . . . . . . . . . . . A A A A A A A    
1166	. . . . . . . . . . . . . . . C C C C C C C    
1167	. . . . . . . . . . . . . . . T T T T T T T    
1168	. . . . . . . . . . . . . . . G G G G G G A    
1169	. . . . . . . . . . . . . . . C C C C C C C    
1170	. . . . . . . . . . . . . . . C C C C C C C    
1171	. . . . . . . . . . . . . . . G G G G G G G    
1172	. . . . . . . . . . . . . . . T T T T T T T    
1173	. . . . . . . . . . . . . . . T T C T T T T    
1174	. . . . . . . . . . . . . . . . . . . . . T    
1175	. . . . . . . . . . . . . . . . . . . . . C    
1176	. . . . . . . . . . . . . . . . . . . . . A    
1177	. . . . . . . . . . . . . . . . . . . . . G    
1178	. . . . . . . . . . . . . . . . . . . . . C    
1179	. . . . . . . . . . . . . . . . . . . . . T    
1180	. . . . . . . . . . . . . . . . . . . . . T    
1181	. . . . . . . . . . . . . . . . . . . . . C    
1182	. . . . . . . . . . . . . . . . . . . . . C    
1183	. . . . . . . . . . . . . . . . . . . . . T    
1184	. . . . . . . . . . . . . . . . . . . . . T    
1185	. . . . . . . . . . . . . . . . . . . . . T    
1186	. . . . . . . . . . . . . . . . . . . . . C    
1187	. . . . . . . . . . . . . . . . . . . . . A    
1188	. . . . . . . . . . . . . . . . . . . . . A    
1189	. . . . . . . . . . . . . . . . . . . . . C    
1190	. . . . . . . . . . . . . . . . . . . . . A    
1191	. . . . . . . . . . . . . . . . . . . . . A    
1192	. . . . . . . . . . . . . . . . . . . . . C    
1193	. . . . . . . . . . . . . . . . . . . . . G    
1194	. . . . . . . . . . . . . . . . . . . . . C    
1195	. . . . . . . . . . . . . . . . . . . . . C    
1196	. . . . . . . . . . . . . . . . . . . . . T    
1197	. . . . . . . . . . . . . . . . . . . . . G    
1198	. . . . . . . . . . . . . . . . . . . . . T    
1199	. . . . . . . . . . . . . . . . . . . . . T    
1200	. . . . . . . . . . . . . . . . . . . . . C    
1201	. . . . . . . . . . . . . . . . . . . . . C    
1202	. . . . . . . . . . . . . . . . . . . . . C    
1203	. . . . . . . . . . . . . . . . . . . . . T    
1204	. . . . . . . . . . . . . . . . . . . . . C    
1205	. . . . . . . . . . . . . . . . . . . . . T    
1206	. . . . . . . . . . . . . . . . . . . . . C    
1207	. . . . . . . . . . . . . . . . . . . . . C    
1208	. . . . . . . . . . . . . . . . . . . . . A    
1209	. . . . . . . . . . . . . . . . . . . . . A    
1210	. . . . . . . . . . . . . . . . . . . . . C
```
